# FGF10 maintains distal lung bud epithelium and excessive signaling leads to progenitor state arrest, distalization, and goblet cell metaplasia

**DOI:** 10.1186/1471-213X-8-2

**Published:** 2008-01-10

**Authors:** Pia Nyeng, Gitte A Norgaard, Sune Kobberup, Jan Jensen

**Affiliations:** 1 Cleveland Clinic Foundation, Lerner Research Institute, Stem Cell Biology and Regenerative Medicine, 9500 Euclid Avenue, Cleveland Ohio, USA; 2 Barbara Davis Center for Childhood Diabetes, University of Colorado Health Sciences Center, 1775 N Ursula St. B140, 80045 Aurora, CO, USA

## Abstract

**Background:**

Interaction with the surrounding mesenchyme is necessary for development of endodermal organs, and Fibroblast growth factors have recently emerged as mesenchymal-expressed morphogens that direct endodermal morphogenesis. The fibroblast growth factor 10 (*Fgf10*) null mouse is characterized by the absence of lung bud development. Previous studies have shown that this requirement for *Fgf10 *is due in part to its role as a chemotactic factor during branching morphogenesis. In other endodermal organs *Fgf10 *also plays a role in regulating differentiation.

**Results:**

Through gain-of-function analysis, we here find that FGF10 inhibits differentiation of the lung epithelium and promotes distalization of the embryonic lung. Ectopic expression of FGF10 in the lung epithelium caused impaired lung development and perinatal lethality in a transgenic mouse model. Lung lobes were enlarged due to increased interlobular distance and hyperplasia of the airway epithelium. Differentiation of bronchial and alveolar cell lineages was inhibited. The transgenic epithelium consisted predominantly of proliferating progenitor-like cells expressing Pro-surfactant protein C, TTF1, PEA3 and Clusterin similarly to immature distal tip cells. Strikingly, goblet cells developed within this arrested epithelium leading to goblet cell hyperplasia.

**Conclusion:**

We conclude that FGF10 inhibits terminal differentiation in the embryonic lung and maintains the distal epithelium, and that excessive levels of FGF10 leads to metaplastic differentiation of goblet cells similar to that seen in chronic inflammatory diseases.

## Background

The lung forms as two evaginations from the ventral foregut at E9.5, a few days after the initial anterior to posterior specification of the uniform gut tube takes place. Sequential branching of the epithelium forms an intricate tree of airways with a distinct axis of proximal to distal differentiation, and a coordinated formation of blood vessels at the distal end. An understanding of the budding process that leads to epithelial branching is quite advanced. Organ culture experiments have shown that branching morphogenesis depends on the presence of lung mesenchyme, which induces branching in tracheal epithelium [1], and that the mesenchymal-expressed fibroblast growth factor 10 (FGF10) can substitute for mesenchyme [2]. The importance of FGF10 for lung development is demonstrated by the fact that *Fgf10 *null mice die at birth due to numerous defects, one of them being the absence of lung buds [3,4]. Using lung explant culture Bellusci et al and Park et al demonstrated that FGF10 acts as a chemoattractant for the epithelium in lung buds in vitro [2,5]. *Fgf10 *expression studies suggest that FGF10-signaling plays an iterative role during lung branching morphogenesis in vivo, as *Fgf10 *is expressed in a dynamic pattern at the tip of each forming bud [2]. Although it is still unknown precisely how this *Fgf10 *expression pattern is controlled, factors that regulate *Fgf10 *expression in the lung include *Fgf9 *[6], *Tgf-beta *[7], *Shh *[2] and *Bmp4 *[8], and interplay between the budding epithelium (expressing SHH and BMP4) and the mesenchyme causes a rapid downregulation of *Fgf10 *as soon as budding is initiated.

In vivo studies of the mechanism by which *Fgf10 *regulates development of the lung are limited, but a study employing transgenic overexpression of *Fgf10 *by promoter elements from Clara cell secretory protein (*Ccsp*) or surfactant protein C (*SftpC*) genes demonstrated a pronounced prenatal and postnatal effect of *Fgf10 *overexpression. Transgenic expression could be detected starting from E15.5 after embryonic induction and resulted in perturbed branching morphogenesis and formation of adenomatous malformations in E17.5 lungs, demonstrating that localized dynamic expression is the key to proper branching. Postnatal overexpression of *Fgf10 *led to tumor formation and differentiation of epithelial tumor cells into a distal type II alveolar cell like phenotype. Affected areas regressed into proximal cell types upon FGF10 cessation indicating that the differentiated distal cells in this transgenic setting required ongoing FGF10 signaling to be maintained [9]. This result indicates that *Fgf10 *may play a role in selectively regulating differentiation of the two different pulmonary cell lineages; the proximal conducting airways (bronchi and bronchioles) and the distal respiratory airways (alveolar epithelium). The bronchial epithelium contains Clara cells, ciliated cells and neuroendocrine cells, while the alveolar epithelium consists of type I and type II alveolar cells. Since most of these cell types are specified already before birth, and ectopic FGF10 has the potential to balance cell types in favor of distal cells postnatally, it is possible that *Fgf10 *plays a role in this prenatal specification, although this question has not yet been addressed.

FGF10 has been shown to regulate prenatal differentiation in other endodermal organs and act as a progenitor maintenance factor. In the pancreas, *Fgf10 *null mice are depleted of progenitor cells leading to arrest of growth and differentiation of the epithelium [10], while overexpression causes expansion of progenitor cells and attenuation of differentiation [11,12]. A similar function is seen in the gastrointestinal tract, as *Fgf10 *null mice exhibit a decrease in dividing progenitor cells [13], and ectopic overexpression leads to attenuation of differentiation in a lineage specific manner [14].

The apparent dichotomy of an established role of FGF10 as a chemoattractant in the lung versus a progenitor maintenance factor in the gastrointestinal organs might be rooted in lack of available information due to the dramatic early phenotype in the lungs of *Fgf10 *null mice. Indeed, a recent paper has demonstrated that dosage of FGF10 in the lungs is critical for progenitor cell amplification. Hypomorphic lungs exhibit severe hypoplasia most likely due to insufficient progenitor cell maintenance and reduced vascular development [15].

During the course of our analyses of the role of *Fgf10 *on more posterior endoderm (stomach, pancreas and gut), based on a model of ectopic expression of *Fgf10 *using the supposedly pancreas/duodenal/gastric specific *Pdx1 *(Pancreatic and Duodenal homeobox 1) promoter element, we surprisingly detected a dramatic effect on lung development. The transgenic model allowed us to address the hypothesis that *Fgf10 *controls differentiation in the embryonic lung epithelium and functions in lung progenitor maintenance. We here demonstrate that the endogenous PDX1 protein is expressed in developing lung at reduced levels compared to the previously described expression in posterior stomach, pancreas and anterior duodenum [14,16], and we find elevated level of *Fgf10 *mRNA and protein in lungs of the pPdx1-*Fgf10 *model from E14.5. We observe effects on multiple aspects of lung development upon ectopic presence of *Fgf10 *within the developing lung epithelium: Our studies confirm that epithelial branching is impaired in *Fgf10 *overexpressing lung lobes [9]. Additional effects in the embryonic lung include increased cell proliferation, distalization of the epithelium, attenuation of terminal differentiation, and goblet cell metaplasia. Our work using a gain-of-function approach suggests that the tight control of *Fgf10 *expression observed in the lung is important in controlling the balance between proliferation and differentiation as well as cell lineage determination.

## Results

### FGF10^FLAG ^is expressed in lung lobes of pPdx1-*Fgf10*^FLAG ^mice

**Figure 1:**
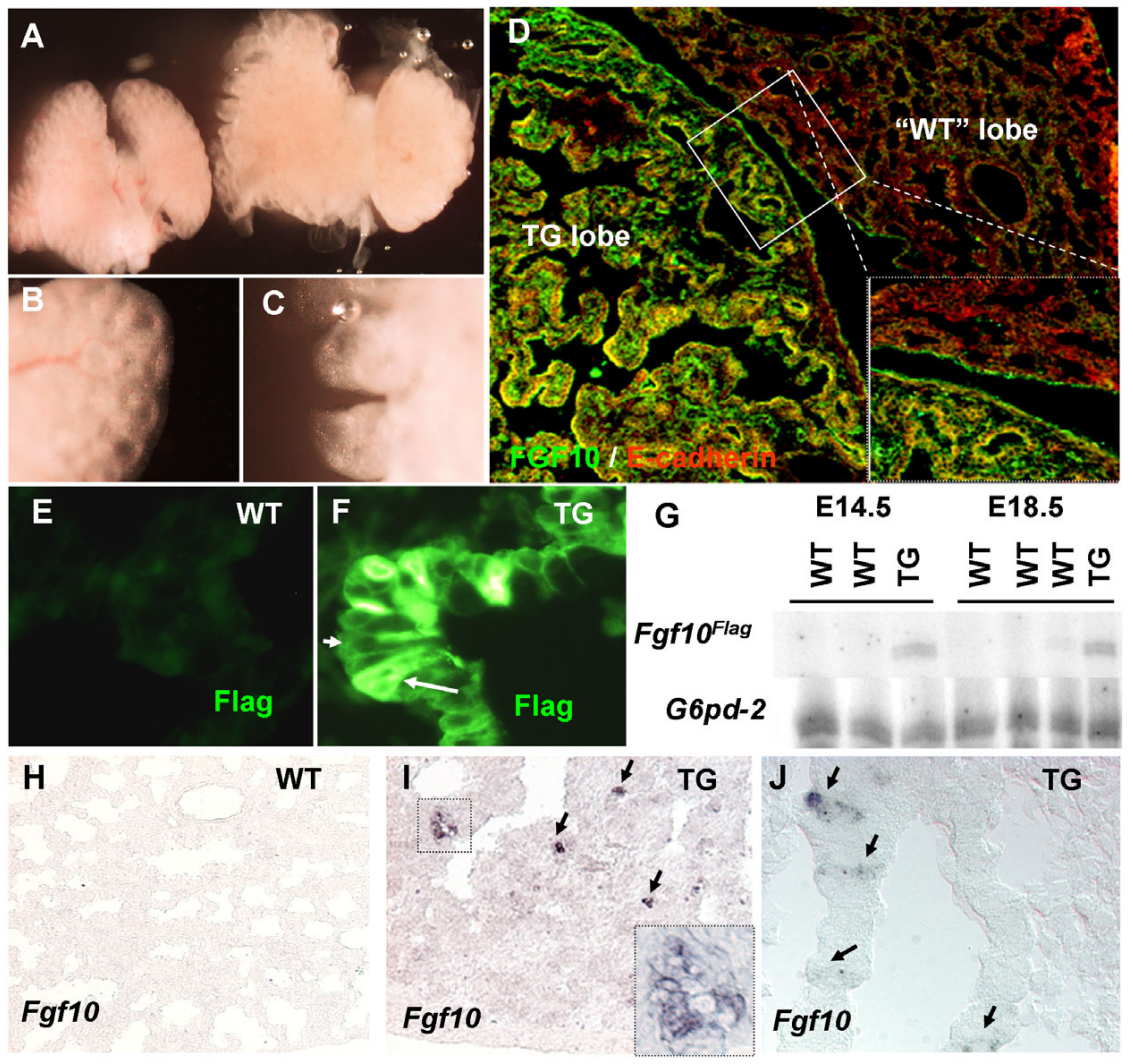
**Expression of pPdx1-*Fgf10*^FLAG^**. A: Comparison of normal lung, left, and transgenic, right at E18.5. The transgenic lungs are larger, and exhibit an enlarged branching pattern, with increased lobular distance. B: higher magnification of normal lung, showing the regularly spaced terminal branches, with only minor grooves between lobules. C: transgenic lung at similar magnification, showing enlarged grooves between terminal branches, where individual lobules are covered with a significantly increased mesenchymal tissue layer. D-J: Lung Expression of pPdx1-*Fgf10*^FLAG^. D: FGF10 staining on E18.5 TG embryo showing the lobe specific expression of the transgene in this embryo. E: FLAG staining on E18.5 WT and F: TG. Arrow in F indicates a cell with high cytoplasmic FLAG staining while the arrowhead indicates a cell with only membrane staining. G: 26 cycle MPX RT-PCR of E14.5 and E18.5 lung (unable to detect *Fgf10 *in lung epithelium of E12.5) using primers for *Fgf10 *and *G6pd-2 *(Glucose-6-phosphate dehydrogenase gene). H-J: *Fgf10 *ISH on TG lung and WT littermate, arrows point to *Fgf10 *positive epithelial cells. H-I: E18.5 WT (H) and TG (I). J: E14.5 TG.

Transient transgenic mice expressing a Flag-tagged version of mouse *Fgf10 *under the *Pdx1 *promoter elements were obtained by oocyte injection as previously described [11]. Although this *Pdx1 *promoter is thought to be specific to the pancreas, duodenum and posterior stomach, we found a profound phenotype in the lung in approximately half of the transient transgenic (TG) mice, causing perinatal lethality. Initial assessment of pPdx1-*Fgf10*^FLAG ^embryos with malformed lungs revealed enlargement of one or more lung lobes, and an increase in the distance between terminal branches (Fig 1A–C). The phenotype varied between individual mice; in two of the transgenic mice analyzed by histochemistry the entire lung was aberrantly formed, while the other two embryos had abnormal formation of only one of the right lobes or the entire left lobe. Immunohistochemistry for FGF10, using an antibody that only detects high amounts of protein, as well as an antibody for the Flag-tag demonstrated that epithelial cells in these lung lobes contained transgenic FGF10, while the normal lobes did not (Fig 1D and 1E–F). Some cells displayed abundant FGF10 expression in the entire cytoplasm (Fig 1F arrow), suggesting that these cells synthesize FGF10, while the majority of cells only stained for FGF10 in the cell membrane (Fig 1F arrowhead). In order to validate the production of transgenic FGF10 in the lung itself, we performed in situ hybridization for *Fgf10 *and low cycle multiplex (MPX) RT-PCR for *Fgf10*^FLAG ^mRNA using *G6pd-2 *as a control. MPX RT-PCR for the transgenic construct showed that *Fgf10*^FLAG ^mRNA was detectable in the TG lung (Fig 1G). In situ hybridization with a short detection time showed *Fgf10 *in the epithelium of the transgenic lung lobes of E14.5 and E18.5 embryos (Fig 1H–J), confirming ectopic presence of *Fgf10*. The highest *Fgf10 *mRNA expression was confined to small clusters of cells in the E18.5 embryos (Fig 1I insert) and to isolated cells at E14.5 (Fig 1J). Variation in expression of *Fgf10 *in pancreas and stomach due to mosaicism in these embryos has been reported before [11,14], in all cases, FGF10 protein expression was widespread due to the secreted nature of the protein (Fig 1D).

**Figure 2:**
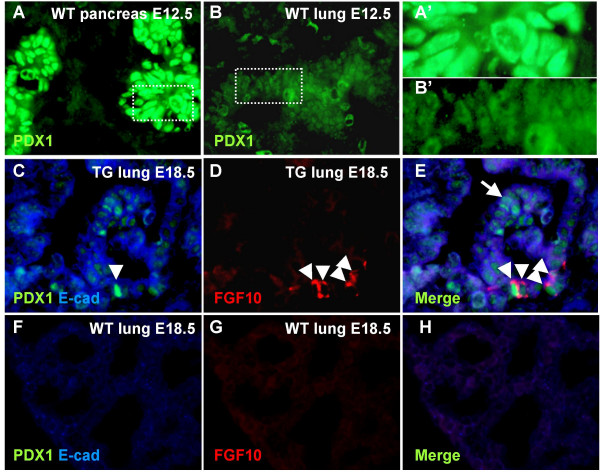
**PDX1 expression**. A-B: PDX1 immunostaining on E12.5 wildtype embryos. A: Strong PDX1 expression in pancreas. B: Much weaker, but still detectable nuclear PDX1 expression in the lung bud. A': Magnification from A. B': Magnification from B. C-H: PDX1 immunostaining on E18.5 TG and WT lung. C: PDX1 and E-cadherin IHC demonstrates that PDX1 is upregulated in the TG lung. D: FGF10 IHC on TG lung. E: Merge of PDX1 and FGF10 IHC demonstrates that not all PDX1 positive cells express cytoplasmic FGF10 (arrow), but all FGF10 producing cells express PDX1(arrowhead). F: PDX1 and E-cadherin IHC on WT lung. G: FGF10 IHC on WT lung. H: merge of F and G.

The observed phenotype and the expression of *Fgf10*^FLAG ^mRNA and protein in the transgenic lung demonstrate that the *Pdx1 *promoter construct must be active in this tissue. This was surprising given the previously reported very accurate replication of endogenous *Pdx1 *expression conferred by the construct in the pancreas, duodenum and posterior stomach [11,14]. As the *Pdx1 *promoter fragment used is limited to app. 4.5 kb upstream regulatory sequence, lung expression may be explained by absence of regulatory elements involved in repression of the *Pdx1 *promoter outside the distal foregut domain. However, it was also possible that *Pdx1 *is normally expressed in lung at a low level and would therefore not have been previously detected. We tested this latter hypothesis by performing an analysis of PDX1 expression throughout embryonic development. Our results revealed that PDX1 protein is present in the early lung buds at E12.5, albeit at a very low level compared to pancreatic and duodenal expression (Fig 2A–B). We were unable to detect any PDX1 expression in E14.5 (results not shown) and E18.5 (Fig 2F) wildtype lungs, but in transgene expressing lung lobes significant PDX1 protein expression was maintained in clusters of epithelial cells (Fig 2C). These lobes exhibited the transgenic phenotype in the entire lobe, likely attributed to the secreted nature of FGF10. Not all PDX1 expressing cells displayed high levels of cytoplasmic staining for FGF10, but all FGF10 secreting cells were positive for PDX1 (Fig 2E). These observations argue that FGF10 is capable of autocrine maintenance of cells with *Pdx1 *expression, otherwise lost during development.

### Disruption of branching morphogenesis and bronchiole formation

**Figure 3:**
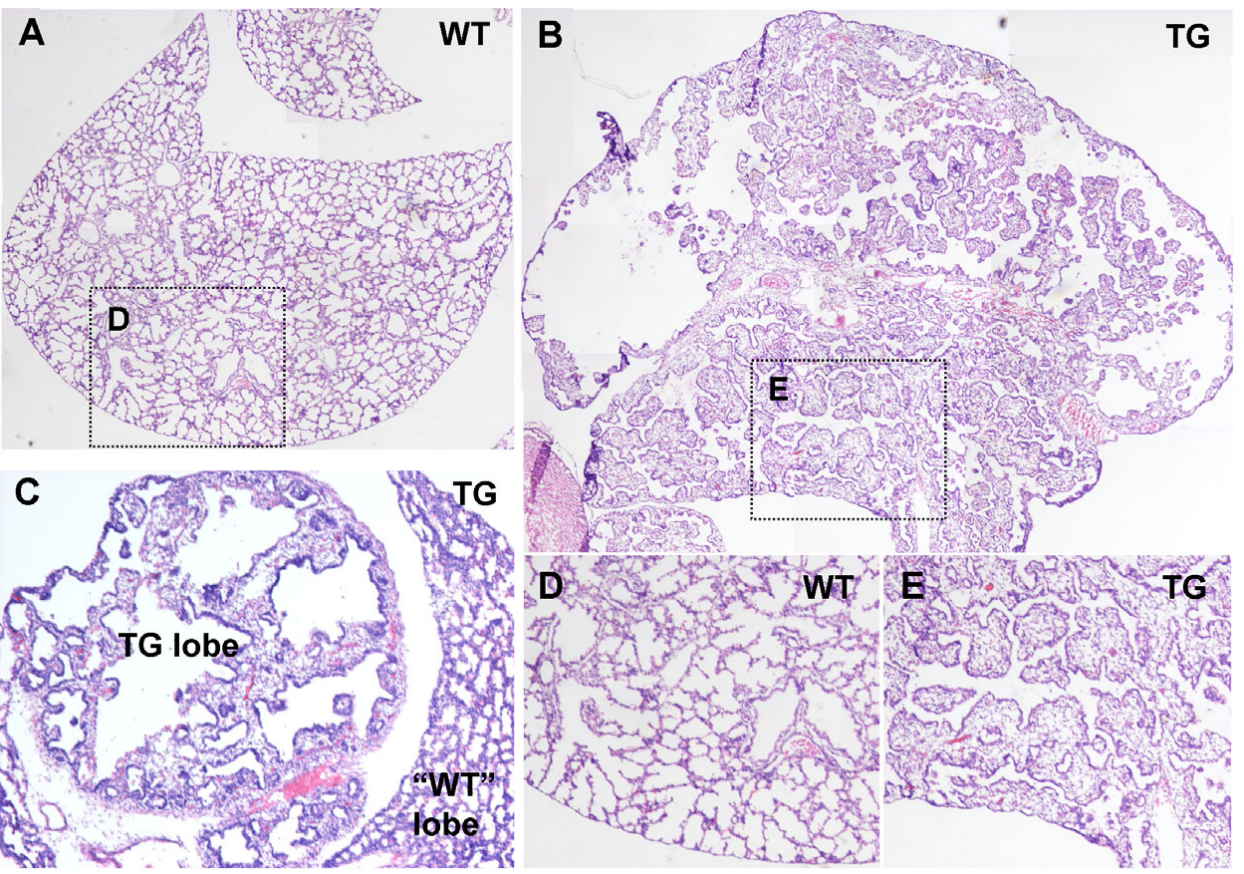
**Lung development is highly abnormal in transgenic embryos**. Hematoxylin and Eosin staining. A: Wildtype E18.5 lung lobe. B: Transgenic E18.5 lung lobe. Transgenic embryos exhibited highly abnormal lung development with disruption of branching morhogenesis leading to formation of empty lumens and dense tissue in place of alveolar sacks. The alveolar epithelium appeared undifferentiated without the typical flattening usually seen at this stage. Proximal bronchi development had occurred to some degree, but distal bronchioles were absent. C: One of two embryos that expressed *Fgf10*^FLAG ^in a single lobe only. Surrounding lobes expressed no transgene and appeared normal, thus providing and excellent comparison for the transgenic phenotype. D: Magnification from A. E: Magnification from B. The lung lobe shown in C and the transgenic lung represented in B are shown throughout the paper, as they were representative for the phenotypes.

Histological examination of transgenic E18.5 lungs revealed that the increased size observed was due to hyperplastic growth as well as formation of large empty lumens surrounded by a mesenchymal layer (Fig 3B and 3C). Branching morphogenesis was disrupted leading to formation of dense areas of tissue composed of mesenchyme and epithelium, somewhat resembling the pseudoglandular stage (E9.5–15.2). Proximal bronchi were formed to some degree, but terminal bronchioles were absent (compare figure 3D and 3E). These results support previous findings [9] that *Fgf10 *overexpression in the lung is lethal at birth due to pulmonary defects including perturbed branching morphogenesis.

In embryos with only one malformed lobe, the surrounding lobes appeared normal (Fig 3C). Throughout this study we show data obtained from one embryo with a single malformed lobe (represented in figure 3C) as well as data obtained from one transgenic embryo with the entire lung malformed and one wildtype littermate embryo (represented in figures 3B and 3A), as each was representative for their phenotype. As we cannot exclude that wildtype-like lobes in the transgenic embryos have been exposed to ectopic FGF10 early in development, the TG lung lobes displaying wildtype morphology are labeled "WT" on the figures also containing TG lung lobes with a pronounced phenotype.

### FGF10 overexpression results in lung hyperplasia due to increased cell proliferation

**Figure 4:**
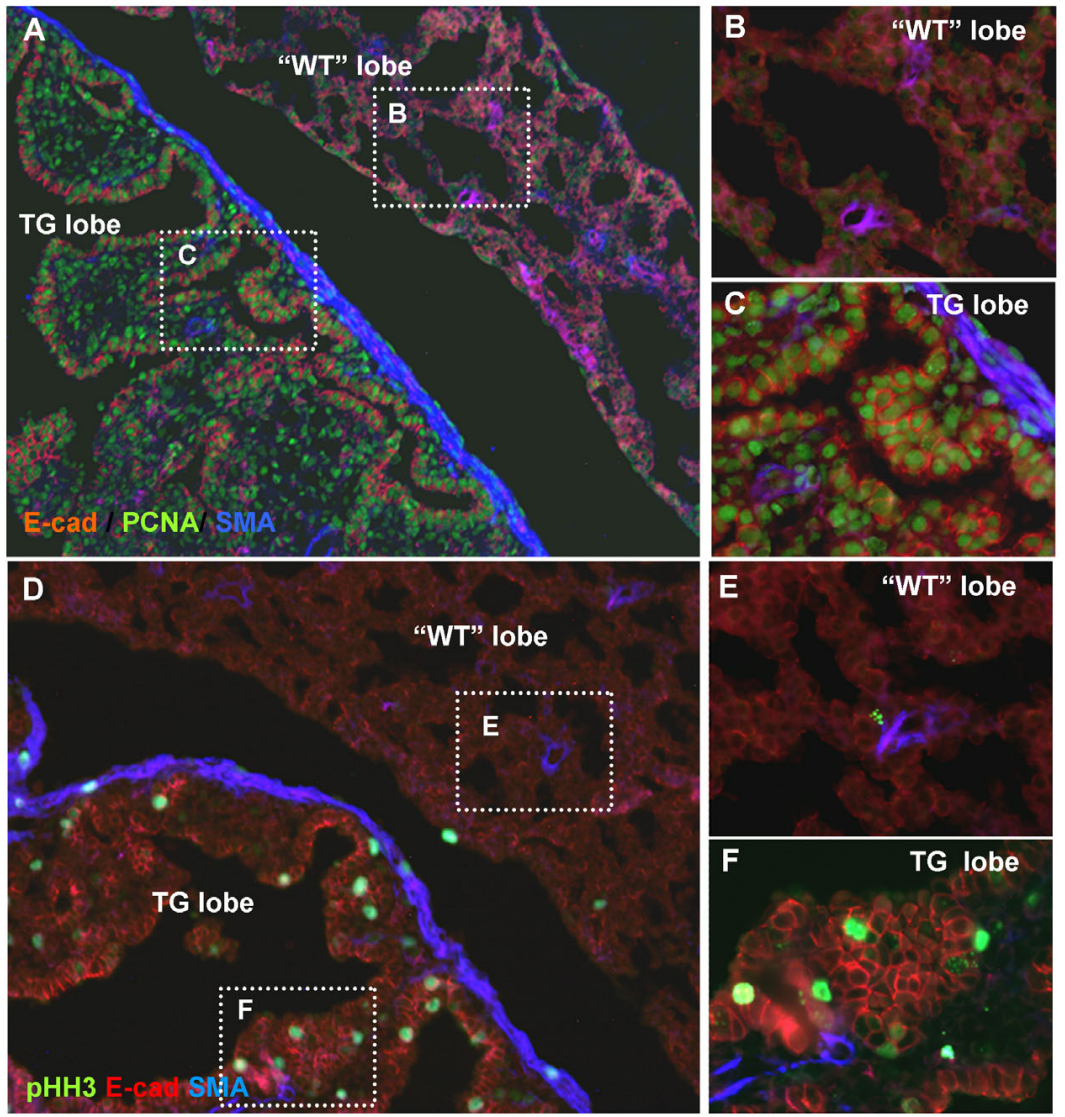
**Increase in number of cells undergoing proliferation**. A-C: PCNA, E-cadherin and smooth muscle actin IHC shows that more cells undergo mitosis in the TG lobe mesenchyme and epithelium, and that a rim of smooth muscle cells surround the TG lobe. A: TG lung with non *Fgf10*^FLAG ^expressing lobe ("WT") and transgene expressing lobe (TG). B and C are magnified from A. D-F: Phospho-histone H3, E-cadherin and smooth muscle actin IHC. D: TG lung with non *Fgf10*^FLAG ^expressing lobe ("WT") and transgene expressing lobe (TG). Many cells are in M-phase in the TG lobe, but very few in the "WT" lobe. E and F are magnified from J.

The number of cells undergoing mitosis at E18.5 in the alveolar tissue is normally negligible (Fig 4A–B and 4D–E), although the entire epithelium in the bronchi is still positive for Proliferating Cell Nuclear Antigen (PCNA) (results not shown). In contrast, the majority of cells were PCNA positive throughout the transgenic lung lobes, in mesenchyme and epithelium (Fig 4A and 4C), demonstrating that transgenic lung lobes are still in a proliferative state. Indeed, as judged from phosphorylated Histone H3 (pHH3) staining, many cells were in M-phase in the TG lobe, but very few in the "WT" lobe (Fig 4D–F). These results support previous findings that FGF10 stimulates pulmonary proliferation in vitro [2] and correspond with the reduction of epithelial cell proliferation seen in *Fgf10 *hypomorphic mice [15].

### FGF10 promotes formation of smooth muscle

FGF10 is expressed in parabronchial smooth muscle progenitors and mice hypomorphic for *Fgf10 *and mice with a *FgfR2b *splicing defect exhibit a reduction in smooth muscle cells [15,17]. We therefore proceeded to analyze expression of smooth muscle actin by immunohistochemistry. Parabronchial smooth muscle cells seemed unaltered in those cases where proximal bronchi did form, but unlike wildtype lungs, transgenic lungs had developed a layer of smooth muscle actin positive cells surrounding the perimeter of the lung lobe (Fig 4A,D and see also Fig 6A and 6D). A few cells within this band were actively dividing (pHH3 positive) (Fig 4D).

### Attenuation of alveolar differentiation and retention of markers of the undifferentiated distal buds

**Figure 5:**
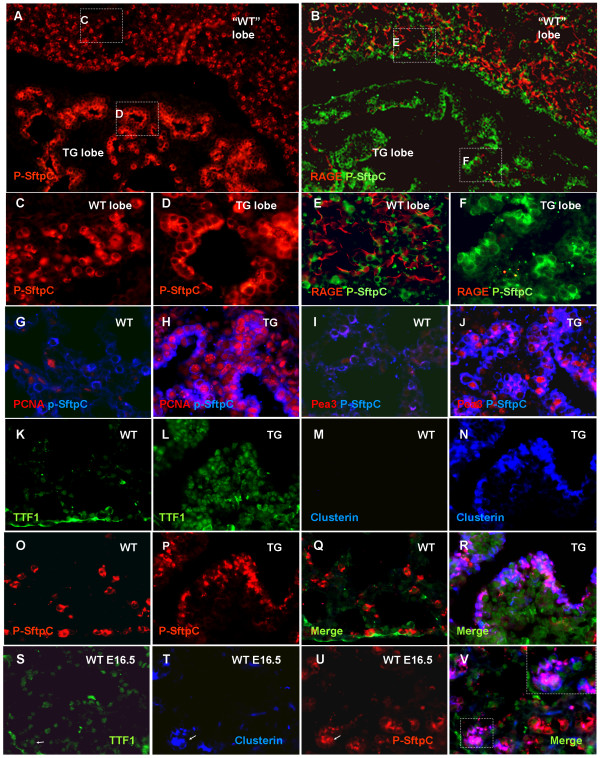
**Alveolar differentiation is attenuated and early distal bud markers upregulated**. A-B: TG lung with non FGF10^FLAG ^expressing lobe ("WT") and transgene expressing lobe (TG). A: Pro-surfactant protein C IHC. B: Pro-surfactant protein C and RAGE IHC. C-D: High magnification from A. E-F: High magnification from B. G-H: PCNA and pro-surfactant protein C IHC. G: WT lung, with no PCNA positive alveolar type II cells. H: TG lung with double positive cells. I-J Pea3 and pro-surfactant protein C IHC. I: WT lung with no Pea3 positive alveolar type II cells. J: TG lung with several double positive cells. K-L: TTF1 IHC on WT (K) and TG (L) show expression mainly in TG cells. M-N: Clusterin IHC on WT (M) and TG (N) show expression only in TG cells facing the lumen. O-P: pro-surfactant protein C IHC on WT (O) and TG (P). Q: merge of K, M and O. R: Merge of L, N and P shows triple positive cells. S-V: E16.5 WT lung S: TTF1 IHC. T: Clusterin IHC. U: Pro-surfactant protein C IHC. V: merge of S-U demonstrates that TTF1, clusterin and p-SftpC are co-expressed in the distal bud (arrowhead in S-V and inset in V) at E16.5.

The high number of cells still undergoing mitosis at E18.5 indicated that transgenic pulmonary cells had not differentiated to the same extent as wildtype. Pro-surfactant protein C is expressed as early as E11.5 in the murine lung progenitor cells [18] and is also expressed by alveolar type II cells developing before birth. This protein was expressed in both TG and WT lungs, but was expressed in cuboidal cells arranged in a simple epithelium in the transgenic lobes (Fig 5A and 5D) as opposed to the rounded solitary alveolar type II cells in WT lungs (Fig 5A and 5C). The majority of transgenic epithelial cells were pro-surfactant protein C positive. Analysis of expression of the receptor for advanced glycation end-products (RAGE), a marker for alveolar type I cells (gas exchange cells) [19,20] revealed an almost complete absence in TG lungs (Fig 5B and 5F). Type I alveolar cells are abundant in E18.5 wildtype lungs and constitute a major part of the alveolar epithelium, with type II alveolar cells occupying the corners between type I cells (Fig 5B and 5E).

The absence of type I cells in the transgenic lobes and the presence of pro-surfactant protein C expressing cells that did not resemble mature type II cells, suggested that differentiation was inhibited. Further characterization of the pro-surfactant protein C expressing cells showed that these cells were actively dividing (Fig 5G and 5H), and that they expressed the ETS factor PEA3 (ETV4) (Fig 5J), which is a FGFR-signaling target and a marker of distal bud cells [21]. This transcription factor was only expressed in a few cells in the wildtype alveolar tissue, and was not expressed in type II cells (Fig 5I).

In order to determine if FGF10 overexpression had resulted in arrest of pulmonary cells in a state resembling the undifferentiated distal bud cells, as suggested by PEA expression, we analyzed the presence of several other markers that characterize these cells. *Ttf1 *(*Nkx2.1*) encodes a lung-specific homeodomain factor that is expressed from E11.5 in the lung bud epithelium and after birth in type II cells [22]. We detected presence of TTF1 in transgenic epithelial cells, but only in a few peripheral wildtype cells (Fig 5K–L). Clusterin is a glycoprotein that is associated with cell damage in adult cells and with differentiation during development. It is expressed transiently during branching morphogenesis in the lung and is localized in the epithelial cells of the distal buds [23]. Clusterin was not expressed in WT E18.5 lung, but was expressed in transgenic epithelial cells facing the lumen (5M-N). TTF1, clusterin and pro-surfactant protein C were co-expressed by the transgenic epithelial cells (Fig 5R), similar to what is seen in the distal tips during the late pseudoglandular stage (E9.5–E15.2) (Fig 5S–V and additional file 1). The observed expansion of the expression pattern of several early distal markers indicate that *Fgf10 *overexpression leads to distalization of the entire embryonic lung, similar to what has previously been found in postnatal lung tumors [9]. The absence of mature distal type I and type II cells furthermore indicate that the transgenic cells, although specified to a distal fate, are arrested in a progenitor-like state.

### Bronchi and bronchiole formation and differentiation are disrupted by FGF10

**Figure 6:**
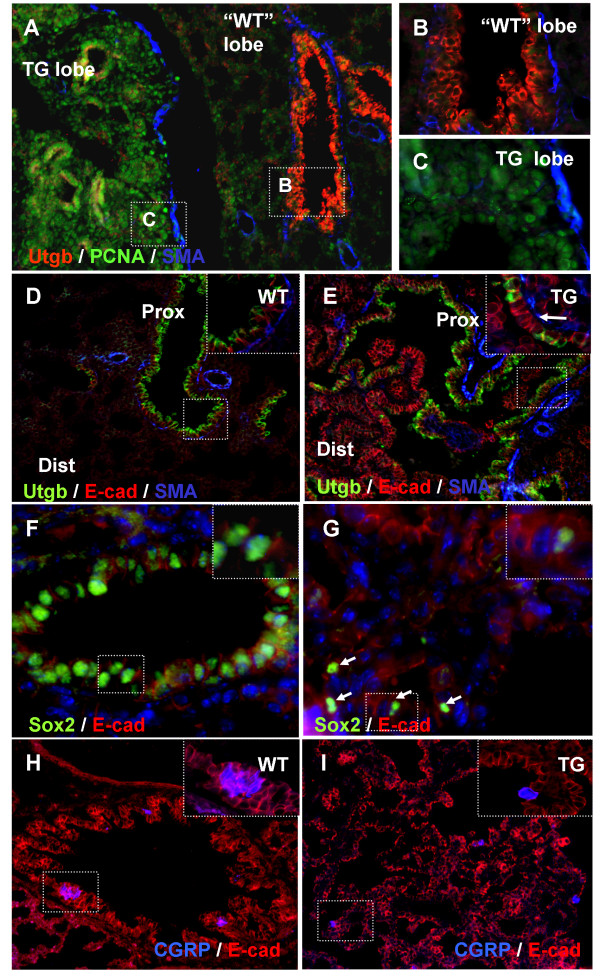
**Attenuation of mature lung bronchial cells**. IHC on E18.5 embryos. A-C: Uteroglobin, PCNA and smooth muscle actin IHC. Showing distal part of transgenic lung which has one *Fgf10*^FLAG ^negative untransformed lobe ("WT" lobe), and one lobe expressing *Fgf10*^FLAG ^(TG). No uteroglobin positive Clara cells are seen in the distal TG lung (A and C), while bronchioles in the "WT" lobe contain Clara cells (A and B). D-E: Uteroglobin, E-cadherin and smooth muscle actin IHC oriented with most proximal end in upper right corner. D: WT proximal lung. E: TG proximal lung. Arrow in inset points to Uteroglobin negative cells. F-G: SOX2 and E-cadherin IHC on distal lung. F: WT bronchiole with SOX2 positive ciliated cells. G: TG lung with a few scattered Sox2 positive cells. H-I: CGRP and E-cadherin IHC. H: WT bronchus with clusters of PNECs. I: TG lung with scattered CGRP positive cells.

Initial assessment of the phenotype revealed that terminal bronchioles were missing, while proximal bronchi development had taken place to some degree, but had differentiation of the bronchial cell lineages taken place? Analysis for expression of uteroglobin, a Clara cell marker, revealed that, as expected from the lack of terminal bronchiole development, there were no Clara cells in the distal lung (Fig 6A and 6C). Proximal bronchi development was also perturbed as the number of cells expressing uteroglobin was reduced compared to wildtype (Fig 6D,E, arrow in E insert points to a gap in expression). Sox2 is expressed in ciliated cells of wildtype trachea and bronchi ([24] and 6F), and expression of this marker was greatly reduced by FGF10. Ciliated cells were found in the most proximal areas, but were similarly reduced as the Clara cells (data not shown), while they were almost absent from more distal areas (Fig 6G). Only a few scattered ciliated cells remained in the distal epithelium suggesting that bronchiole development was aborted (Fig 6G). Interestingly, one particular bronchial lineage descendant was capable of forming. CGRP expressing pulmonary neuroendocrine cells (PNECs) were still present in the transgenic lung lobes. In the wildtype lung these cells were confined to bronchi and bronchioles and appeared in small clusters (Fig 6H), while they were scattered throughout the transgenic lung (Fig 6I). Our results support the assessment that bronchiole development was terminated, as only PNECs were present in the distal transgenic lung.

### Expression of PDX1 in the lung does not lead to transdifferentiation to intestinal, gastric or pancreatic fates

**Figure 7:**
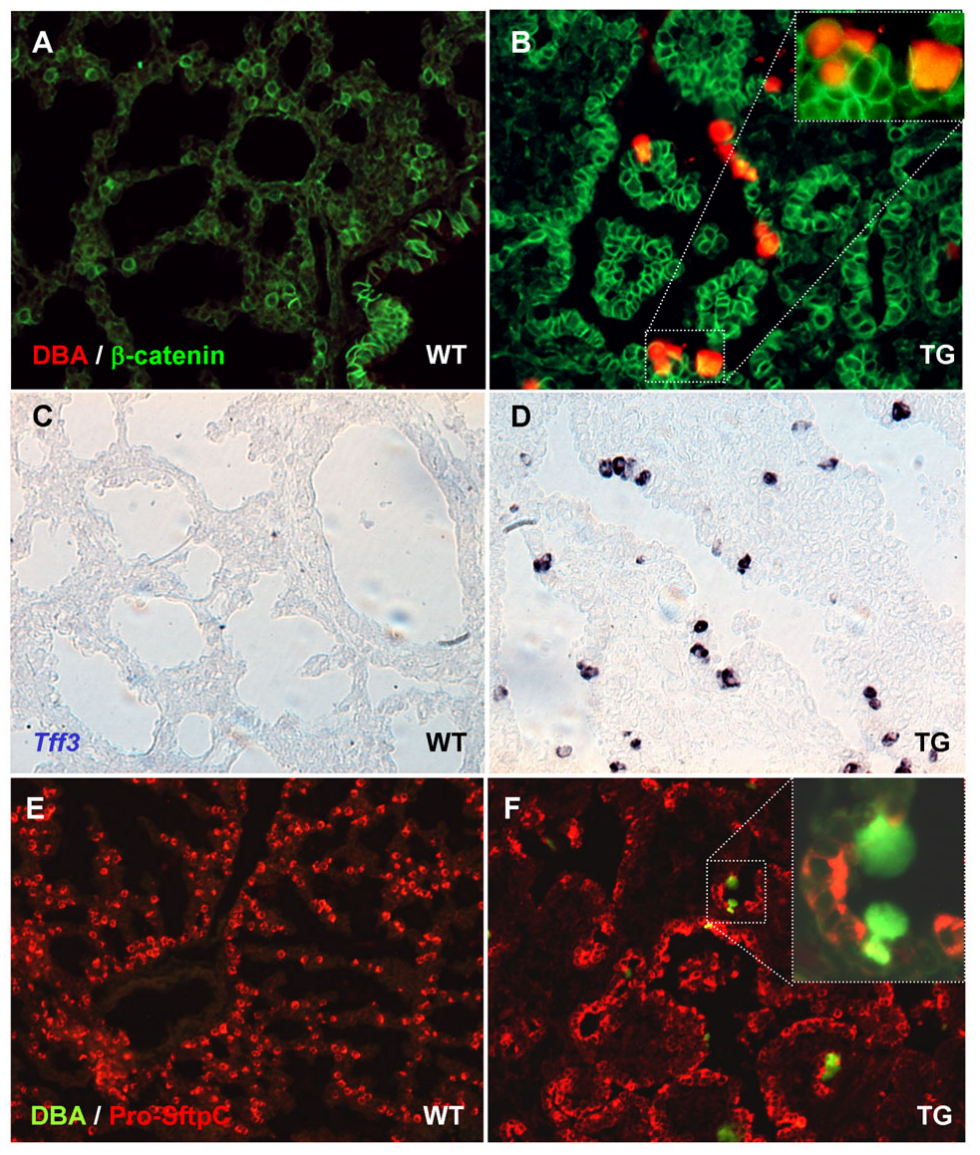
**Goblet cell metaplasia in the alveolar epithelium**. A-B: IHC with Rhodamine-conjugated Dolichos Biflorus Agglutinin (DBA) and Beta-catenin. Goblet cells detected by their DBA reactivity were present in the alveolar epithelium of the transgenic mice (B), while no goblet cells were detected anywhere in the wildtype lungs (A). C-D: ISH using a Trefoil factor 3 probe. No Tff3 was detected in wildtype lung. Goblet cells in the transgenic lung were positive (D). E-F: IHC for pro-Surfactant protein C and fluorescein conjugated Dolichos Biflorus Agglutinin (DBA). Goblet cells did not co-express pro-Surfactant protein C.

The observation that transgenic lungs lacked terminal pulmonary cells lead to speculations of whether transdifferentiation to a pancreatic, gastric or intestinal fate had taken place, due to ectopic expression of FGF10 and PDX1. We performed immunohistochemistry for expression of NKX6.1 (pancreas marker) and insulin, but did not detect expression, indicating that transdifferentiation to a pancreatic fate did not occur (see Additional files 2, figures A-C). CDX2 is expressed throughout the intestine, MATH1 is expressed by intestinal secretory cell lineages, Cryptdin3 by Paneth cells and intestinal fatty acid binding protein (*Fabp2*) by enterocytes. In the transgenic lungs we could not detect CDX2 or *Fabp2 *expression (Additional file 2figures D and J), and only very few *Cryptdin3 *or MATH1 expressing cells (Additional file 2 figures G and D. Insert shows positive cells in Fig G). Sox2 is expressed in the gastric epithelium, throughout the esophagus, and is present in the ciliated cells in the bronchi [24], but very little expression was seen in the transgenic tissue outside of bronchi (Fig 6F–G). Likewise, H/K-ATPase and PepsinogenC, expressed by Parietal and chief cells respectively, were also absent (results not shown). Trefoil factor 1 (*Tff1*) is normally expressed by mucous producing cells in the stomach (Additional file 2, figure O), and is absent from embryonic lung (Additional file 2, figure N). A few *Tff1 *positive cells were found in one out of three transgenic mice (Additional file 2, figure M). *Tff3 *is expressed in intestinal goblet cells, and although not expressed in embryonic lung (Fig 7C), is expressed by pulmonary goblet cells in the tracheal submucosal glands in adult mice [25]. *Tff3 *was expressed in several cells throughout the distal epithelium in all transgenic lobes (Fig 7D). We therefore conclude that transdifferentiation to a gut like fate had not taken place, although limited expression of MATH1 and *Tff1 *as well as high expression of *Tff3 *pointed towards a possible goblet cell metaplasia.

### Embryonic goblet cell metaplasia

Goblet cells are present in only few numbers in submucosal glands of adult mice although more cells can form as a response to inflammation in adult mice and humans, and in some human lung cancers. Goblet cells react with Dolichos Biflorus Agglutinin (DBA) [26], and detection of rhodamine or Cy2 conjugated DBA, as well as PAS/alcian blue histochemistry (data not shown), confirmed that goblet cells were present in the distal epithelium of transgenic but not wildtype lobes (Fig 7A and 7B). No DBA reactivity was found in bronchi or trachea. Since this goblet cell metaplasia had arisen in the prospective alveolar epithelium, we performed a double immunofluorescence analysis for DBA and pro-surfactant protein C. Similar to WT lung, DBA and p-SftpC were not co-expressed (Fig 7F, insert).

### Increased Beta-catenin signaling and *Bmp4 *expression

**Figure 8:**
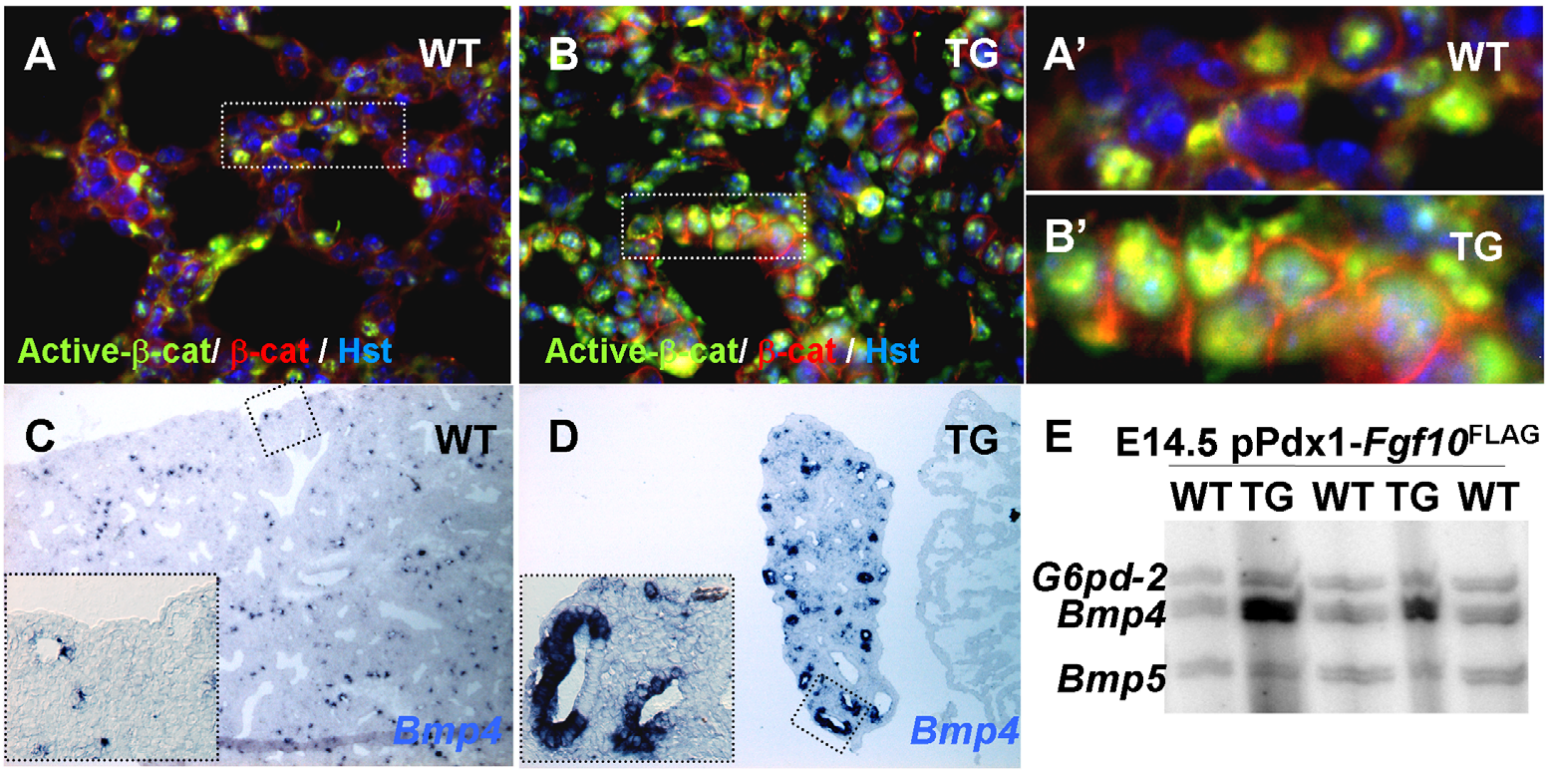
**Beta-catenin activation and upregulation of BMP4 expression**. A-B: IHC using an antibody that is specific for activated beta-catenin dephosphorylated on Ser37 or Thr4, and an antibody that recognizes only membrane bound beta-catenin. Counterstained with Hoechst (hst). A: Wildtype alveolar lung contains scattered cells with activated beta-catenin. B: Beta-catenin is activated in more cells in the transgenic lung. A': magnification from A. B' Magnification from B. In both WT and TG, cells are double positive for activated and membrane-bound beta-catenin, implying that cells retain membrane-bound beta-catenin even when activated. C-D: ISH using a probe for *Bmp4*. *Bmp4 *was expressed in both wildtype (C) and transgenic (D) distal lung epithelium, but transgenic expression was much more widespread. Part of the right atrium (*Bmp4 *negative) is seen to the right hand side of the TG lung lobe in D. E: Multiplex-RTPCR of E14.5 wildtype and transgenic lung using primers for *G6pd-2 *(Glucose-6-phosphate dehydrogenase gene), *Bmp4 *and *Bmp5 *shows upregulation of *Bmp4 *mRNA.

Regulation of transcription through activation of stabilized beta-catenin is necessary for differentiation of distal airways [27], and transgenic expression of an activated form of beta-catenin leads to goblet cell metaplasia and expansion of alveolar type II-like cells [28]. Since goblet cell metaplasia and expansion of surfactant Protein C expressing cells were also observed in the pPdx1-*Fgf10*^FLAG ^model, we next tested whether the observed phenotype could be caused partly through activation of beta-catenin (reviewed in [29]). We analyzed for activation of beta-catenin using an antibody specific for the dephosphorylated Serine37 or Threonine41, which is an activated form [30]. Cells positive for activated beta-catenin were present throughout the epithelium and mesenchyme in both wildtype and transgenic lobes (Fig 8A–B), and more activated cells were present in the distal part of the wildtype lung (data not shown), than the proximal part (Fig 8A). The frequency of activated beta-catenin cells was higher in the transgenic lobes, especially in the proximal lung (Fig 8B) but also in the distal lung (data not shown), suggesting that *Fgf10 *overexpression leads to activation of beta-catenin.

*Bmp4 *is expressed in the distal-most epithelial cells in proximity to the FGF10 expressing mesenchyme, and *Bmp4 *expression is upregulated in response to a FGF10 bead [8]. We therefore analyzed whether *Bmp4 *expression was increased in the pPdx1-*Fgf10*^FLAG ^lung. In situ hybridization revealed that *Bmp4 *was expressed in scattered epithelial cells at E18.5 (Fig 8C), while the number of *Bmp4 *positive cells was increased in transgenic lungs (Fig 8D). This result was confirmed by MPX RT-PCR, where *Bmp4 *but not *Bmp5 *expression was highly upregulated in transgenic lungs (Fig 8E).

These results confirm previous in vitro data and demonstrate that *Fgf10 *overexpression leads to activation of beta-catenin and upregulation of epithelial *Bmp4 *expression, suggesting that these are downstream effectors of *Fgf10 *and could be involved in the observed phenotype. This observation is in agreement with previous reports showing that reductions in FGF-signaling leads to reduced Wnt-signaling [31].

## Discussion

Development of the lung is an intricate process whereby coordination of branching of the epithelium and concomitant differentiation leads to a highly patterned organ. Embryonic lung formation by branching morphogenesis depends on chemotactic attraction by FGF10 secreted from the mesenchyme [2-4]. This is however not the only role of FGF10 during lung development. We here provide evidence that in addition to controlling branching morphogenesis, FGF10 maintains the undifferentiated state of the progenitor like cells of the distal buds, hereby coordinating the two processes.

We show here that PDX1 is expressed by the epithelium of embryonic lung at E12.5, and we employ a *Pdx1 *promoter construct to drive *Fgf10 *expression from this time onwards, as ectopic FGF10 expression presumably allows for maintenance of PDX1 expression. Notwithstanding a possible physiological role of *Pdx1 *in lung development which is not investigated here, the *Pdx1 *promoter driven expression of *Fgf10 *is sufficient to elicit a significant effect on lung development. We confirm previous results showing that overexpression of *Fgf10 *in the embryonic lung epithelium is highly disruptive for branching morphogenesis [9], and demonstrate that the observed enlargement of the lung is due to hyperplastic growth and formation of large internal cavities. We conclude that *Fgf10 *overexpression has a mitogenic effect in vivo similar to what has been previously demonstrated in vitro [2], corresponding with the reduction of epithelial cell proliferation seen in *Fgf10 *hypomorphic mice [15]. Transgenic lungs were covered by a dense layer of smooth muscle, also supporting previous findings by Mailleux et al [17] that FGF10 is required for entry of mesenchymal cells into the parabronchial smooth muscle cell lineage.

Based on previous studies of the role of FGF10 in more posterior endoderm [11,12,14] and the postnatal effect of FGF10 overexpression on differentiation of tumors [9], we wished to explore the hypothesis that *Fgf10 *functions in embryonic lung progenitor maintenance. At E18.5, transgenic pulmonary cells were phenotypically similar to wildtype cells before completion of the canalicular stage (E16.6–17.4). The majority of the transgenic airway cells remained undifferentiated and mitotic at a time when cells outside of the bronchial epithelium normally cease active proliferation and terminal lung cells differentiate. Markers of early distal cells such as TTF1, the FGFR target PEA3 and Clusterin were co-expressed with pro-Surfactant protein C, while markers of differentiated alveolar type I cells and bronchial cells were largely absent. Pro-surfactant protein C is expressed both early in development and in alveolar type II cells, but mature alveolar type II cells do not co-express TTF1 and Clusterin or PEA3. In contrast, these markers were co-expressed in the transgenic lung and in wildtype E16.5 distal bud cells before differentiation. These results demonstrate that ectopic overexpression of *Fgf10 *results in attenuation of differentiation and promotion of a progenitor-like distal cell type. An embryonic progenitor maintenance program such as this is necessary for sufficient expansion of the progenitor pool before differentiation, but can contribute to oncogenesis if reinstated during postnatal life. The previous finding that postnatal *Fgf10 *overexpression induces lung tumors with high expression of TTF1 and SftpC thus corresponds with our findings that FGF10 maintains the distal progenitor-like cells during early embryonic development.

Consistent with the published FGF10 induction of *Bmp4 *expression in lung buds in vitro [8], we observed upregulation of *Bmp4 *expression in the TG lung. It is likely that part of the observed effects are caused by the altered expression of *Bmp4*, as BMP signaling has been shown to be necessary for the morphologically correct development of lung buds [32]. However, BMP4 has an anti-proliferative effect in the lung, suggesting that the proliferating progenitor-like cells seen in the pPdx1-*Fgf10*^FLAG ^lung are maintained by a different mechanism. Our results indicate that this mechanism works through activation of beta-catenin. In previous studies, ectopic expression of an activated form of beta-catenin caused distalization of the proximal airways and goblet cell hyperplasia [28], while ectopic expression of a beta-catenin-Lef1 fusion protein lead to expansion of progenitor cells and intestinal metaplasia of the lung epithelium [33]. The observed difference between the two studies is probably due to binding specificity of Lef1 as opposed to TCF factors. We observe a similar expansion of distal-like progenitor cells, indicating that activation of beta-catenin may be the main mechanism through which FGF10 maintains undifferentiated distal cells. We conclude that the arrested lung phenotype resembles that of the undifferentiated distal lung buds, possibly caused by an increase in *Bmp4 *expression and beta-catenin activation, and appears incapable of normal differentiation under conditions of high FGF10 expression. Consequently, FGF10 elicits lung epithelial progenitor arrest, in a comparable manner to that observed in pancreas [11], stomach [14] and duodenum (Nyeng et al. Manuscript submitted), indicating a conserved role of FGF10 in the endoderm.

In the light of the attenuated lung program, it was possible that transdifferentiation of the lung to a pancreatic, gastric or intestinal fate had occurred as a result of ectopic FGF10 and PDX1 expression. Respecification of endodermal cells has been noted in several cases. Overexpression of *Pdx1 *in the liver of Xenopus tadpoles leads to transdifferentiation into pancreas [34], and forced expression of *Ptf1a *and *Pdx1 *is capable of inducing a pancreatic fate in more anterior endoderm, including stomach, but not lung [35]. We found no evidence of transdifferentiation using pancreatic markers whereas early lung markers such as TTF1 and pro-surfactant protein C were preserved. There was also no evidence of conversion to a gastric fate. Transdifferentiation of lung epithelium to an intestine-like fate as seen in the beta-catenin-Lef1 fusion protein model resulted in expression of Paneth cell markers, *Math1 *and *Cdx1 *among other intestinal genes[33]. Although *Fgf10 *overexpression leads to dephosphorylation and activation of beta-catenin in our model, we did not find evidence to suggest a major transformation towards an intestinal fate. Although we did observe a few MATH1 expressing cells in the lung, and also several goblet cells, which derive from MATH1 expressing cells, the absence of CDX2 and *Fabp2 *argues against major posteriorization of the lung to an intestinal phenotype.

Our observation that goblet cells were present in the prospective alveolar epithelium is indicative of goblet cell metaplasia. Increased presence of goblet cells in the lung is often observed during adult lung pathogenesis such as asthma, COPD and cystic fibrosis. Adult goblet cell hyperplasia has been associated with activation of EGFR resulting in signaling through the RAS-MAP-kinase pathway leading to an increase in expression of genes involved in goblet cell formation (reviewed in [36,37]). It is possible that increased FGFR signaling upstream of the MAP-kinase pathway may trigger the same mechanistic pathway, consistent with our observation of upregulation of PEA3, a target of the MAP-kinase pathway. An alternate mechanism could be that the FGF10 mediated activation of beta-catenin seen in our model lead to goblet cell metaplasia, as expression of an activated form of beta-catenin results in goblet cell hyperplasia of the bronchial epithelium through this mechanism [28,38] Goblet cell metaplasia has been shown to originate from Clara cells (reviewed in [37]) in adult lung pathogenesis. It seems highly unlikely that goblet cells originate from Clara cells in our model, as we did not observe any goblet cells near the bronchial epithelium. Alveolar type II cells function as a progenitor cell for the alveolar cells [39], raising the possibility that metaplastic goblet cells could have differentiated from alveolar type II progenitor cells. We cannot exclude this possibility, although no SftpC and DBA double-positive cells were found. We conclude that *Fgf10 *overexpression leads to embryonic goblet cell metaplasia of the prospective alveolar epithelium, but not conversion to a more posterior endodermal fate. These results demonstrate that, even though *Fgf10 *overexpression perturbs lung morphology and attenuates the lung differentiation program, the lung still retains pulmonary identity.

## Conclusion

FGF10 is normally expressed in proximity to budding epithelium, where it directs branching morphogenesis spatially [2,5]. After a certain degree of completion of the branching process, differentiation occurs into either proximal or distal lung regions, as a response to intra-organ patterning set up early in lung development. Our results here demonstrate that overexpression of *Fgf10 *during embryonic development leads to attenuation of differentiation, distalization of the epithelium and goblet cell hyperplasia. These results suggest that a single molecule, FGF10, is intricately involved in coordinating pulmonary branching morphogenesis and cellular differentiation. In turn, this argues for a possible coordinate regulation of these two processes, which perhaps therefore should not be viewed as separate mechanisms.

## Methods

### Transgenic mice

pPdx1-*Fgf10*^FLAG ^construction and derivation of transgenic embryos by oocyte injection was performed as described in [11], and approved by the UCHSC Animal Care and Use Committee. Day of oocyte injection was counted as day 0.5. A total of 7 E18.5 and 6 E14.5 transient transgenic embryos with a lung phenotype and their wildtype littermates were analyzed in this study. Whole guts from four E18.5 embryos and three E14.5 embryos were fixed in 4% PFA overnight, embedded for freeze sectioning in OCT medium, and sectioned at 6 μm. The remaining embryos were dissected and duodenal RNA was extracted using Trizol. For studies of wildtype E16.5 lung, CD1 mice were mated and the pregnant dam sacrificed on E16.5 (Noon of day of plug was counted as day 0.5).

### Multiplex RT-PCR analysis

cDNA was synthesized from 1 μg of purified RNA and analyzed by semi-quantitative radioactive multiplex RT-PCR (MPX RT-PCR) previously described by [40]. A primer set for glucose-6-phosphate dehydrogenase (*G6pd-2*) (GACCTGCAGAGCTCCAATCAAC and CACGAC CCTCAGTACCAAAGGG) was included in all PCR reactions as an internal control for normalization, and the PCR reactions were run for 26 cycles. A primer pair for the beta-globin tail of the pPdx1-*Fgf10*^FLAG ^construct (GTTGCCGTCAAAGCCATCAAC and CTACTTGTCATCGTCGTCCTT) was used for genotyping and expression analysis. Primers for *Bmp4 *(CTGAGTATCTGGTCTCCGTCC and AAGGCTCAGAGAAGCTGCGGC) and *Bmp5 *(GCAAAAGGAGGCTTGGGAGA and TCGCTAGCCATGGCATTGTA) were used for expression analysis.

### Histology

Immunohistochemistry and in situ hybridization were performed on 6 μm frozen tissue slides fixed in 4% paraformaldehyde as described in [11]. Antigen retrieval was performed for 10 minutes in microwave oven in 0.01 M citrate buffer pH6. Antibodies used in this study include: goat-anti-*Fgf10 *(1:200, Abcam, Cambridge, UK), rabbit-anti-FLAG-HRP (1:1000, Sigma, St. Louis, MO, USA) with TSA, goat-anti-PDX1 (1:2000, CV Wright, Nashville, TN, USA), rat-anti-E-Cadherin (1:200, Zymed/Invitrogen, Carlsbad, CA, USA), mouse-anti-smooth muscle actin (1:500, 1A4, DAKO, Denmark), rabbit-anti-PCNA (1:150, Santa Cruz BT, Santa Cruz, CA, USA), rabbit-anti-phospho-Histone-H3 (1:100, Upstate, VA, USA), mouse-anti-clusterin (1:50, R & D systems, Minneapolis, MN, USA), rabbit-anti-pro-surfactant protein C (1:100, Upstate, Charlottesville, VA, USA), rabbit-a-Uteroglobin (1:1600, Abcam, Cambridge, UK), rabbit-a-RAGE (1:800, Abcam, Cambridge, UK), rabbit-a-beta-catenin (1:200, Neomarkers, Fremont, CA, USA), rabbit-a-PEA3 (1:200, Santa Cruz BT, Santa Cruz, CA, USA) mouse-a-TTF1 (1:800, Affinity Bioreagents, Golden, CO, USA), rabbit-a-SOX2 (1:1000 TSA, Abcam, Cambridge, UK), rabbit-a-CGRP (1:10,000, Sigma, St. Louis, MO, USA) and Rabbit-a-ABC (Active beta-catenin, 1:1000 TSA, Upstate, Charlottesville, VA, USA). TSA signifies whether a tyramide signal amplification kit (Perkin Elmer, CA, USA) was used for detection.

Mature goblet cells were detected by an overnight staining using rhodamine or fluorescein conjugated Dolichos Biflorus Agglutinin (DBA) (Vector Laboratories).

In situ probes where synthesized from mouse *Fgf10 *and *Bmp4 *previously isolated in our lab and cloned in pCR4 plasmids and *Tff1*, *Tff3*, *Cryptdin3 *and *Fabp2 *plasmids (Open Biosystems, Huntsville, AL, USA).

Harris hematoxylin and eosin Y staining was carried out according to standard protocols.

## Authors' contributions

PN participated in conceiving and designing the study, carried out and analyzed the morphological and histological studies and drafted the manuscript. GAN cloned the pPdx1-*Fgf10*^FLAG ^construct and participated in RNA analysis. SK contributed to design of the PDX1 expression experiment, interpretation of data, and revision of the manuscript. JJ participated in conceiving and designing the study, participated in its design, performed RNA analysis and revised the manuscript. All authors read and approved the final manuscript.

## Supplementary Material

Additional file 1 **Co-expression of TTF1, Clusterin and pro-SftpC in the distal bud at E16.5**. IHC on WT E16.5 lung. A: TFF1. B: Clusterin. C: pro-Surfactant protein C. D: Merge of A-C. E-H: Higher magnification of the tip area shown in A-D. E: TFF1. F: Clusterin. G: pro-Surfactant protein C. G: Merge of A-C. Click here for file

Additional file 2 **No evidence of transdifferentiation of the lung epithelium**. IHC and ISH for markers of pancreatic, intestinal and gastric fates. A-C: Nkx6.1, Insulin and E-cadherin IHC of E18.5 TG (A) and WT (B) lung and WT pancreas (C). D-F: CDX2 and MATH1 IHC of E18.5 TG (D) and WT (E) lung and WT intestine (F). A few MATH1 positive cells were found in TG lung (insert). Note that the MATH1 antibody has high background staining in goblet cells. G-I: *Cryptdin3 *ISH of E18.5 TG (G) and WT (H) lung and WT intestine (I). Very few positive cells were found in the TG lung (arrow points to one). J-L: *Fabp2 *(IFABP) ISH of E18.5 TG (J) and WT (K) lung and WT intestine (L). M-O: *Tff1 *ISH of E18.5 TG (M) and WT (N) lung and WT stomach (O). A small area of Tff1 positive cells (M) was found in only one out of three TG mice. Click here for file
