# Supplementary figures and images for: FGF10 maintains distal lung bud epithelium and excessive signaling leads to progenitor state arrest, distalization, and goblet cell metaplasia
Source: BMC Dev Biol. 2008 Jan 10;8:2. doi: 10.1186/1471-213X-8-2 (PMC2263027; doi:10.1186/1471-213X-8-2)

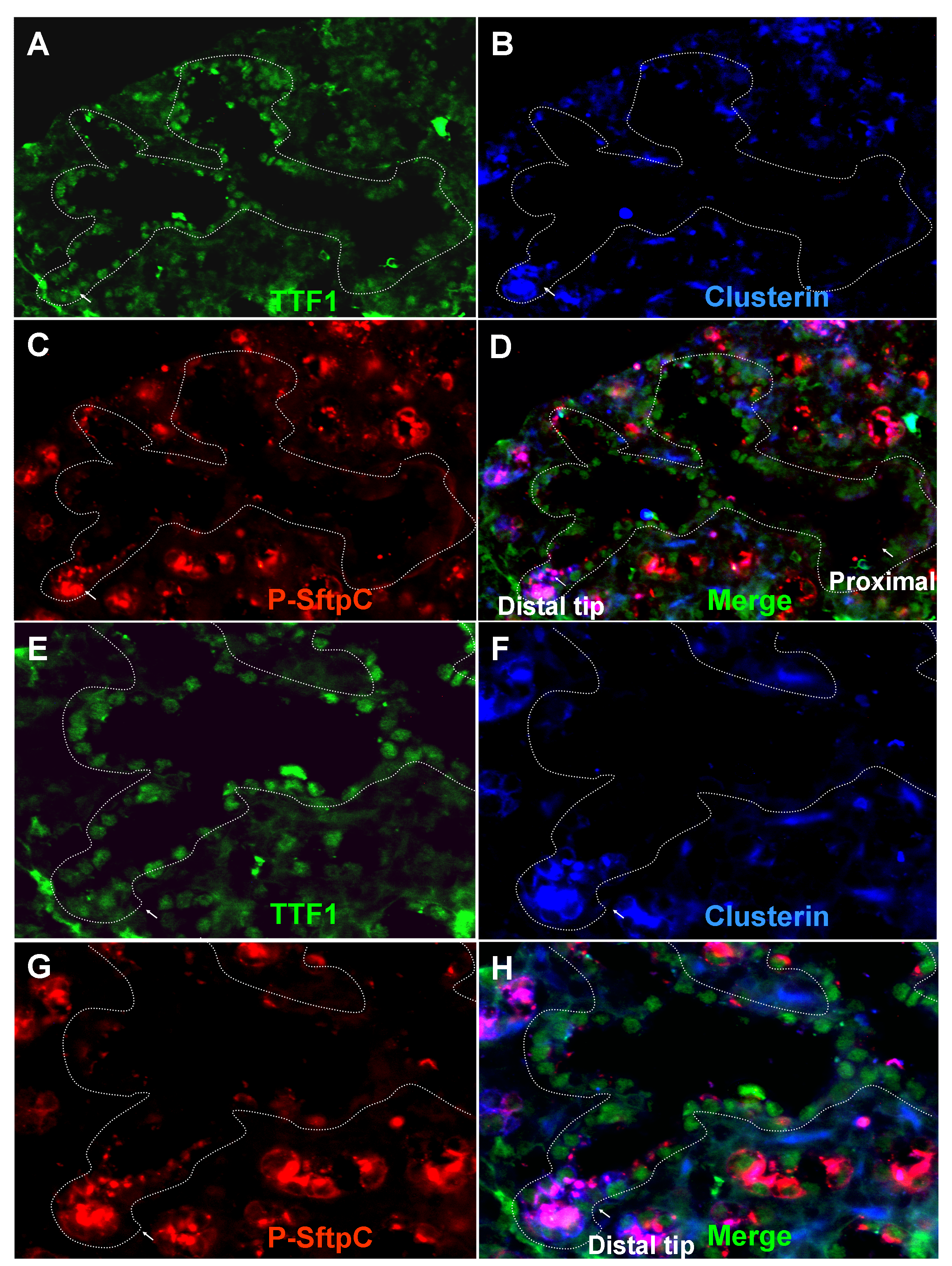

Supplement: Additional file 1 — Co-expression of TTF1, Clusterin and pro-SftpC in the distal bud at E16.5. IHC on WT E16.5 lung. A: TFF1. B: Clusterin. C: pro-Surfactant protein C. D: Merge of A-C. E-H: Higher magnification of the tip area shown in A-D. E: TFF1. F: Clusterin. G: pro-Surfactant protein C. G: Merge of A-C. [file 1471-213X-8-2-S1.TIFF]

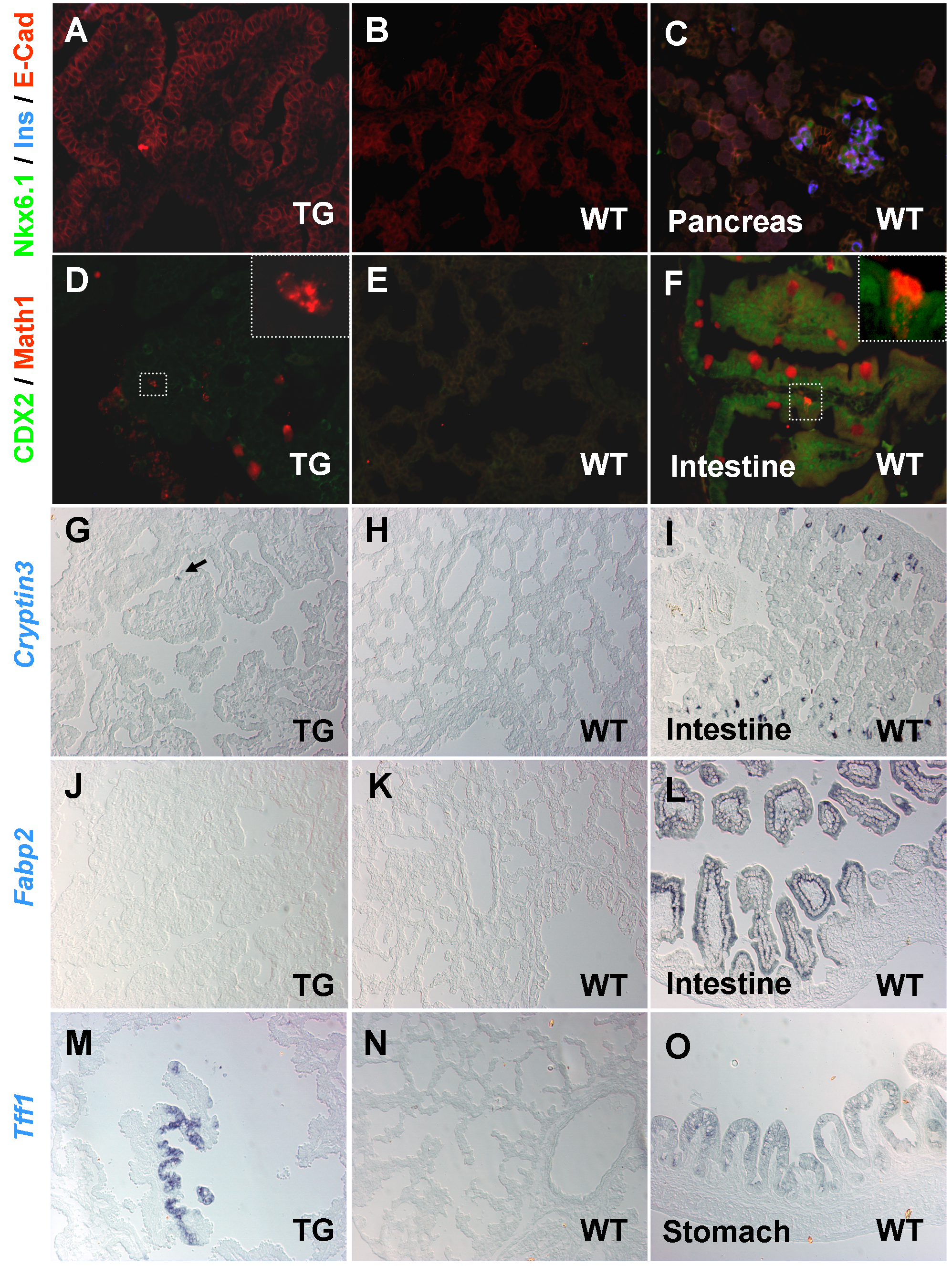

Supplement: Additional file 2 — No evidence of transdifferentiation of the lung epithelium. IHC and ISH for markers of pancreatic, intestinal and gastric fates. A-C: Nkx6.1, Insulin and E-cadherin IHC of E18.5 TG (A) and WT (B) lung and WT pancreas (C). D-F: CDX2 and MATH1 IHC of E18.5 TG (D) and WT (E) lung and WT intestine (F). A few MATH1 positive cells were found in TG lung (insert). Note that the MATH1 antibody has high background staining in goblet cells. G-I: Cryptdin3 ISH of E18.5 TG (G) and WT (H) lung and WT intestine (I). Very few positive cells were found in the TG lung (arrow points to one). J-L: Fabp2 (IFABP) ISH of E18.5 TG (J) and WT (K) lung and WT intestine (L). M-O: Tff1 ISH of E18.5 TG (M) and WT (N) lung and WT stomach (O). A small area of Tff1 positive cells (M) was found in only one out of three TG mice. [file 1471-213X-8-2-S2.TIFF]
